# Application of Microfluidics in Detection of Circulating Tumor Cells

**DOI:** 10.3389/fbioe.2022.907232

**Published:** 2022-05-12

**Authors:** Can Li, Wei He, Nan Wang, Zhipeng Xi, Rongrong Deng, Xiyu Liu, Ran Kang, Lin Xie, Xin Liu

**Affiliations:** ^1^ Affiliated Hospital of Integrated Traditional Chinese and Western Medicine, School of Artificial Intelligence and Information Technology, Nanjing University of Chinese Medicine, Nanjing, China; ^2^ Department of Clinical Medical Engineering, The First Affiliated Hospital of Nanjing Medical University, Nanjing, China; ^3^ Department of Orthopedics, Nanjing Lishui Hospital of Traditional Chinese Medicine, Nanjing, China

**Keywords:** tumor metastasis, circulating tumor cells, microfluidic, biosensor, cancers

## Abstract

Tumor metastasis is one of the main causes of cancer incidence and death worldwide. In the process of tumor metastasis, the isolation and analysis of circulating tumor cells (CTCs) plays a crucial role in the early diagnosis and prognosis of cancer patients. Due to the rarity and inherent heterogeneity of CTCs, there is an urgent need for reliable CTCs separation and detection methods in order to obtain valuable information on tumor metastasis and progression from CTCs. Microfluidic technology is increasingly used in various studies of CTCs separation, identification and characterization because of its unique advantages, such as low cost, simple operation, less reagent consumption, miniaturization of the system, rapid detection and accurate control. This paper reviews the research progress of microfluidic technology in CTCs separation and detection in recent years, as well as the potential clinical application of CTCs, looks forward to the application prospect of microfluidic technology in the treatment of tumor metastasis, and briefly discusses the development prospect of microfluidic biosensor.

## 1 Introduction

Despite decades of deepening understanding of cancer, cancer is still one of the leading causes of death worldwide. It is estimated that the number of cancer cases may increase by 60% in the next two decades. Among them, the growth rate of low—and middle-income countries may be as high as 81% (Wild et al., 2020). The burden of cancer is increasing in all countries, and the cancer burden in low—and middle—income countries is expected to double in the next decade. Without further action, millions of people will die prematurely from cancer in the next decade. Cancer metastasis refers to cells spreading from the primary focus to the distal organs. It is one of the main causes of cancer death (Suhail et al., 2019). Circulating tumor cells (CTCs) are defined as cancer cells that depart from solid tumor lesions and enter the blood, originally discovered by Ashworth (Shen et al., 2017). CTCs are not the only tumor derivative in the circulation, but they contain many metastatic precursors, which is very important for disease progression (Castro-Giner and Aceto, 2020). Clinical circulating tumor cells mainly refer to diverse tumor cells in peripheral blood. Understanding CTCs is helpful to explore the mechanism of primary tumors and metastatic lesions. Early diagnosis of circulating tumor cells (CTCs) can effectively identify patients who need further systemic treatment after initial tumor resection. CTCs detection mainly detects the content of various tumor cells in peripheral blood through capture to detect the changing tendency of CTCs type and quantity and subsequently monitor the dynamic evaluation of tumor treatment effect in real-time. The circulating tumor cells in peripheral blood can be used to help judge the cancer complications of patients. However, the content of CTCs in human blood is infrequent. The contents of red blood cells, white blood cells, and platelets can reach 5 × 10^9^/ml, 4 × 10^6^/ml, and 3 × 10^8^/ml, while CTCs are usually only 0–10/ml (Yu M. et al., 2013). Moreover, tumor cells can constantly transform their characteristics through epithelial mesenchymal transformation and interstitial epithelial-transformation in metastasis. Due to its scarcity, heterogeneity and the interference of complex matrix in blood, the precise detection of CTCs has become an enormous issue.

Currently developed CTCs detection methods, including immunofluorescence (Ramirez et al., 2019; Lin and Chang, 2021), fluorescence *in situ* hybridization (Cheng et al., 2019), reverse transcription-polymerase chain reaction (RT-PCR) (Yang et al., 2017; Tong and Wang, 2019), real-time fluorescence quantitative PCR (Guo et al., 2015), flow cytometry (Galanzha and Zharov, 2013; Ruiz-Rodríguez et al., 2021), immunofluorescence *in situ* hybridization and immunohistochemical staining (Yu et al., 2020; Guo et al., 2019; Wu et al., 2020a), are challenging to meet the requirements of direct detection in detection limit and sensitivity. Therefore, some sample pretreatment methods are usually used to separate and enrich CTCs before detecting CTCs in peripheral blood. In the process of sample pretreatment to realize the separation and enrichment of CTCs, as these methods are discontinuous, it is inevitable to cause the loss of cells in adsorption, elution, and transfer. Additionally, the scarcity of CTCs can easily lead to false-negative results. Moreover, most CTCs detection technologies are time-consuming, require skilled operators and high-tech instruments. Moreover, the detection of CTCs is still challenging due to their low concentration and heterogeneity in blood samples. Therefore, there is an urgent need to develop novel technologies to make the separation and detection of CTCs more convenient, accurate, and noninvasive.

In recent years, microfluidic technology has attracted considerable interest in CTCs detection. Microfluidic technology is characterized by a micro-manufacturing structure, which usually manipulates the fluid with high flux and sensitivity on the micron scale (Cao et al., 2021). With the remarkable progress of micro-machining methods, the microfluid platform has significant advantages such as low cost, good micro-structure, reduced sample consumption, rapid fluid processing, good detection sensitivity, and so on, and is applied to the primary and applied research of oncology (Lin et al., 2020; Pei et al., 2020a). Microfluidic technology makes it possible for rapid and reliable sample separation and high selectivity and sensitivity detection of CTCs. This paper looks forward to the microfluidic CTCs detection system, which is significant for biomedicine and its application in early cancer diagnosis.

## 2 Microfluidic Technologies for CTCs Separation

Microfluidic technology is a new technology used in the primary and applied research of cancer metastasis for decades. A microchannel with a small size is used to accurately control a small volume of liquid or process multiple samples in an integrated bioreactor simultaneously. Compared with traditional methods, it has the advantages of automatic operation, reasonable sensitivity, and throughput, which makes it possible to construct structures on the cell scale. In the past decade, microfluidic platforms based on functional microchannel have been developed to separate CTCs. As a miniaturized analysis, it realizes the one-step process of sample collection, loading, separation, and analysis to significantly reduce the processing time and improve the opportunity to capture CTCs. A microfluidic platform can hinder the interaction between cell and antibody by accurately controlling the direction and speed of fluid flow, which directly impacts the capture efficiency. In addition, it is a simple tool to integrate other technologies/materials (such as ceramics, metals, and polymers) to improve the analysis efficiency of CTCs.

With the growth of solid tumors and specific changes in the surrounding microenvironment, some tumor cells will obtain abnormal activity ability, that is, epithelial-mesenchymal transformation (EMT). These cells shed from the primary tumor and find a new foothold in the body. These tumor cells will be brought to various body parts through the blood system or lymphatic system, and the tumor cells shed through the blood circulation are CTCs. When they reach an appropriate target, they will become malignant reproductive machines. This is the hematogenous metastasis of the tumor from the primary site to the secondary tumor. Therefore, after obtaining the patient’s blood, we first need to separate and enrich the CTCs, analyze the characteristics of these CTCs, and then give the appropriate treatment strategy. However, the content of CTCs in the human circulatory system is shallow (Zou and Cui, 2018). There are only 1∼10 CTCs per ml of the whole blood in patients with tumor metastasis. Therefore, to realize the detection of CTCs, sorting, and enrichment are crucial steps. CTCs’ separation and enrichment will directly affect subsequently detection effect. Therefore, CTCs sorting and enrichment with high purity, high sensitivity (without losing CTCs), fast and high cell activity is the focus and difficulty of CTCs clinical application.

The enrichment of CTCs can be divided into the positive enrichment method of capturing CTCs and the negative enrichment method of removing leukocytes. The positive enrichment method mainly includes affinity and physical enrichment methods. The affinity enrichment method mainly utilizes a specific antibody to combine with tumor cell surface antigen to enrich CTCs specifically. The physical enrichment method mainly screens out CTCs according to their physical characteristics, such as size, density, mechanical and dielectric properties. Due to the slight size difference between leukocytes and CTCs, leukocytes are often the chief interference factor in sorting CTCs in blood. Therefore, leukocytes can be selectively isolated to achieve the purpose of CTCs enrichment, that is, the negative enrichment method.

### 2.1 Positive Enrichment

#### 2.1.1 Enrichment Based on Biological Affinity

The affinity enrichment method mainly separates target cells through the antigen expression on the cell surface, tissue-specific membrane antigens or peptides, and aptamers to capture CTCs.

##### 2.1.1.1 EpCAM Specific Recognition

**FIGURE 1:**
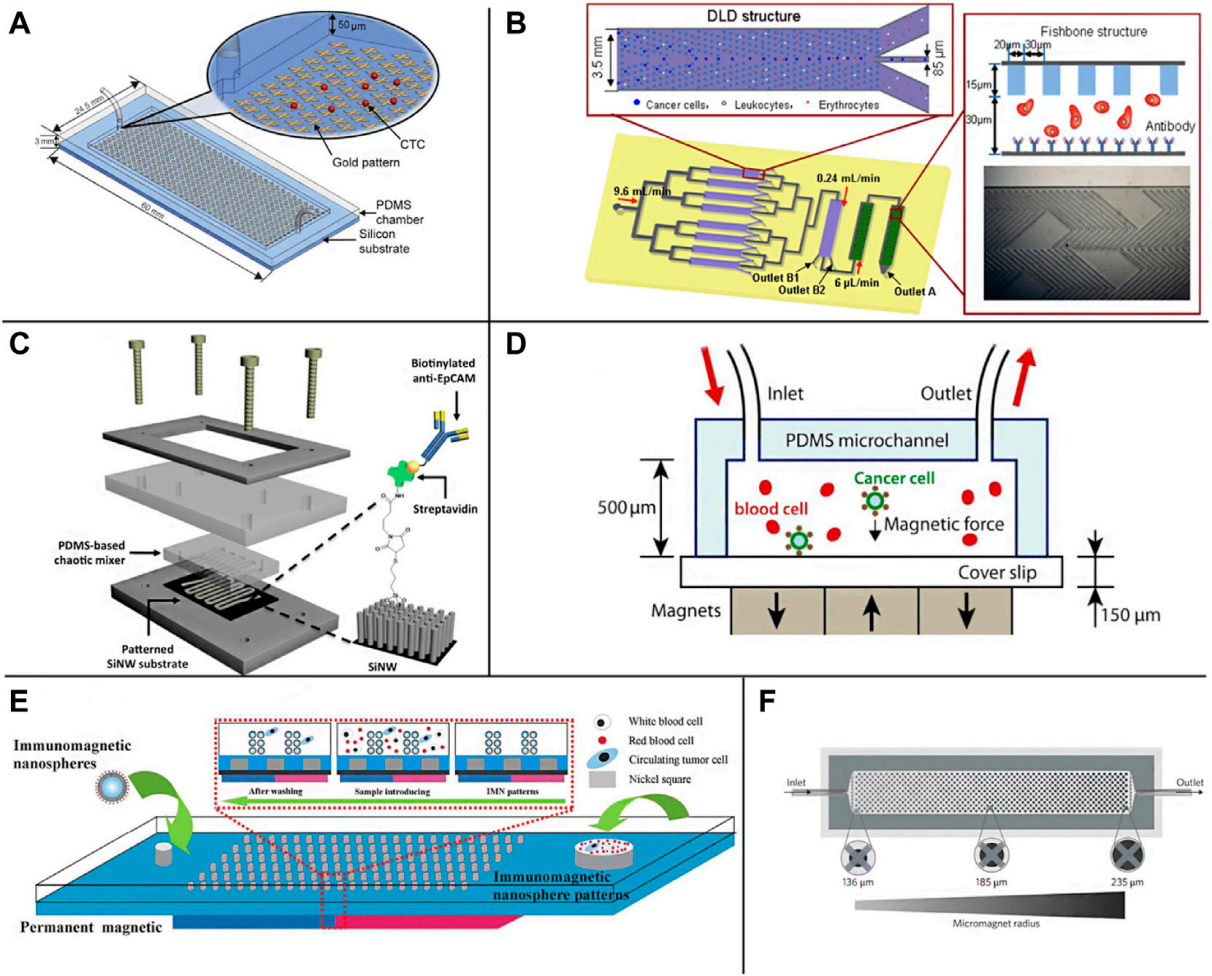
Schematic illustration of the EpCAM specific recognition chips. **(A)** The GO chip (Yoon et al., 2013); **(B)** The deterministic lateral displacement (DLD) chip (Liu et al., 2013); **(C)** The NanoVelcro CTC chip (Lu et al., 2013); **(D)** The microchip designed for immunomagnetic detection of cancer cell (Hoshino et al., 2011); **(E)** The magnetic nanospheres (MNs) based microfluidic device (Tang et al., 2016); **(F)** The wavy-HB microfluidic device (Shi et al., 2017)

CTCs can be divided into epithelial CTCs, mesenchymal CTCs, and mixed phenotype CTCs (Pan et al., 2019), in which epithelial markers are expressed on normal epithelial cells and epithelial tumors but not on interstitial leukocytes. Therefore, they are often distinguished between cancer cells and normal blood cells. Epithelial cell adhesion molecule (EpCAM) is a transmembrane glycoprotein expressed in most solid cancers, so it is one of the most widely used surface markers for CTCs enrichment (Eslami-S et al., 2020; Ahmed et al., 2017; Thege et al., 2014). The EpCAM antibodies can be immobilized on the surface of microchannels, micropores, or other nanostructures to achieve positive capture of CTCs. In 2007, the Toner group reported the first Immunocapture platform CTCs chip, which consists of a series of EpCAM coated micropores and can separate CTCs from whole blood with high sensitivity and high activity. It can be used to capture CTCs from peripheral blood of patients with lung, breast, prostate, pancreatic, and colon cancer metastases (Nagrath et al., 2007). Since then, the microfluidic platform based on EpCAM has developed rapidly. Subsequently, the above group developed a herringbone chip (HB chip), whose unique structure maximizes the collision between cancer cells and the EpCAM coating surface in the microchannel (Stott et al., 2010). Further, Nagrath et al. designed graphene oxide nanosheets on the EpCAM antibody coating for positive CTCs selection (Figure 1A). The improved CTCs chip obtained an average of 73% CTCs capture efficiency from whole blood samples of lung, breast, and pancreatic cancer patients (Yoon et al., 2013). However, a technical challenge of the microfluidic platform is that blood cells pass through the platform in a straight-line streamline with a low Reynolds number, resulting in limited interaction between CTCs and antibodies coated on the surface of the microchannel, thus reducing the capture efficiency. Although the microcolumn array in the CTCs chip developed by Nagrath et al. effectively destroys the laminar flow to improve the interaction between CTCs and antibodies, it is still challenging to capture and target CTCs at different heights along the microcolumn. Therefore, geometric enhancement attracted people’s attention to increase the contact probability between cells and antibody functionalized surfaces. For example, the Soper group proposed a curved microchannel structure coated with EpCAM antibodies to improve the isolation performance of CTCs (Jackson et al., 2014). In addition, an integrated microfluidic platform composed of a triangular microcolumn array allows CTCs to be captured with continuous throughput, efficiency, and purity (Liu et al., 2013) (Figure 1B). Combining the deterministic lateral displacement (DLD) chamber and the anti-EpCAM based capture method, more than 90% capture rate and 90% purity of live CTCs were observed in the added blood samples.

Due to the high surface area volume ratio, compatible capture and release ability, nanomaterials provide a high collision probability for antibodies and CTCs (Park et al., 2017; Wu C. et al., 2019; Shi et al., 2022; Yi et al., 2022; Liu et al., 2022; Liu J. et al., 2021), which can help improve the sensitivity and specificity of capture, and have attracted extensive attention in the separation of CTCs (Wongkaew et al., 2018; Wu and Qu, 2015; Zhu G. et al., 2021; Naskar et al., 2020; Chandankere et al., 2020). So far, carbon nanotubes (CNTs) (Cho H. et al., 2018; Zhang P. et al., 2019; Poudineh et al., 2017), graphene oxide (GO) (Chen et al., 2012; Yoon et al., 2013; Yu X. et al., 2013; Yoon et al., 2016), gold nanoparticles (AuNP) (Park et al., 2017), nanocolumns (Lin et al., 2014; Shi et al., 2022) and TiO_2_ nanofibers (Zhang et al., 2012) have been widely used in affinity group capture methods (Zhao et al., 2016; Cai et al., 2017; Wongkaew et al., 2018). NanoVelcro CTCs chip is a representative nanostructure microfluidic platform. It uses siliceous nanowire substrate (SINS), so that the interaction between SINS and cells can be similar to Velcro (Wang et al., 2011; Jan et al., 2018). The results demonstrate that compared with a planar siliceous substrate, the capture efficiency of anti-EpCAM coated SINS is significantly improved. Fan group has developed a microfluidic platform with gold nanoparticles assembled with multivalent DNA aptamers to efficiently separate CTCs from blood (Sheng et al., 2012). In addition, the Zhao group also developed an antibody functionalized electrospun TiO2 nanofiber (TiNF) matrix for CTCs capture (Zhang et al., 2012). Lu et al. introduced a nanovelcro CTCs chip containing silicon nanowires and a pattern substrate to enumerate CTCs in prostate cancer (Lu et al., 2013) (Figure 1C). They proved its clinical utility in continuous CTCs enumeration of prostate cancer patients and the effectiveness of continuous CTCs enumeration in monitoring cancer progression.

Immunomagnetic enrichment is also a widely used CTCs separation method in a microfluidic platform, which enriches CTCs from blood cells using magnetic particles labeled with anti-EpCAM. With the introduction of a microchip based on Polydimethylsiloxane (PDMS), the Hoshino group proposed an immunomagnetic-based microchip to capture CTCs under a magnetic field (Figure 1D), which combines the dual advantages of magnetophoresis enrichment and microfluidic technology (Hoshino et al., 2011). Continuous operation and improved throughput make PDMS-based immunomagnetic microchannel promising to capture cells. Zhang group has developed a microfluidic device based on magnetic nanospheres (MNS) to capture tumor cells (Figure 1E). The device integrates the functions of the magnetic microfluidic chip and immunomagnetic nanospheres (IMN), forming a new and stable IMN mode (Tang et al., 2016). Liu group has developed a microfluidic device with a wavy HB structure (Figure 1F). Under the external magnetic field, the magnetic particles of anti-EpCAM coating are fixed on the wavy HB surface to capture tumor cells (Shi et al., 2017). Poutine et al. used magnetic grading cytometry to analyze CTCs according to the surface expression phenotype of CTCs. Whole blood samples were processed using microfluidic chips, and then CTCs subsets were captured by controlling magnetic field strength and fluid flow rate based on the number of magnetic nanoparticles labeled on a single cell. The higher EpCAM expressing cells are captured in the higher linear velocity region, while the lower EpCAM expressing cells with fewer magnetic nanoparticles are captured in the lower linear velocity region (Poudineh et al., 2017). By using magnetic nanoparticles to separate and release recovered CTCs, the loss of live CTCs can be reduced without adding biotin. At the same time, this technology can reduce the damage to CTCs in the isolation process and maintain the high throughput of capture performance (Qian et al., 2015).

##### 2.1.1.2 Tissue Specific Membrane Antigen Recognition

**FIGURE 2:**
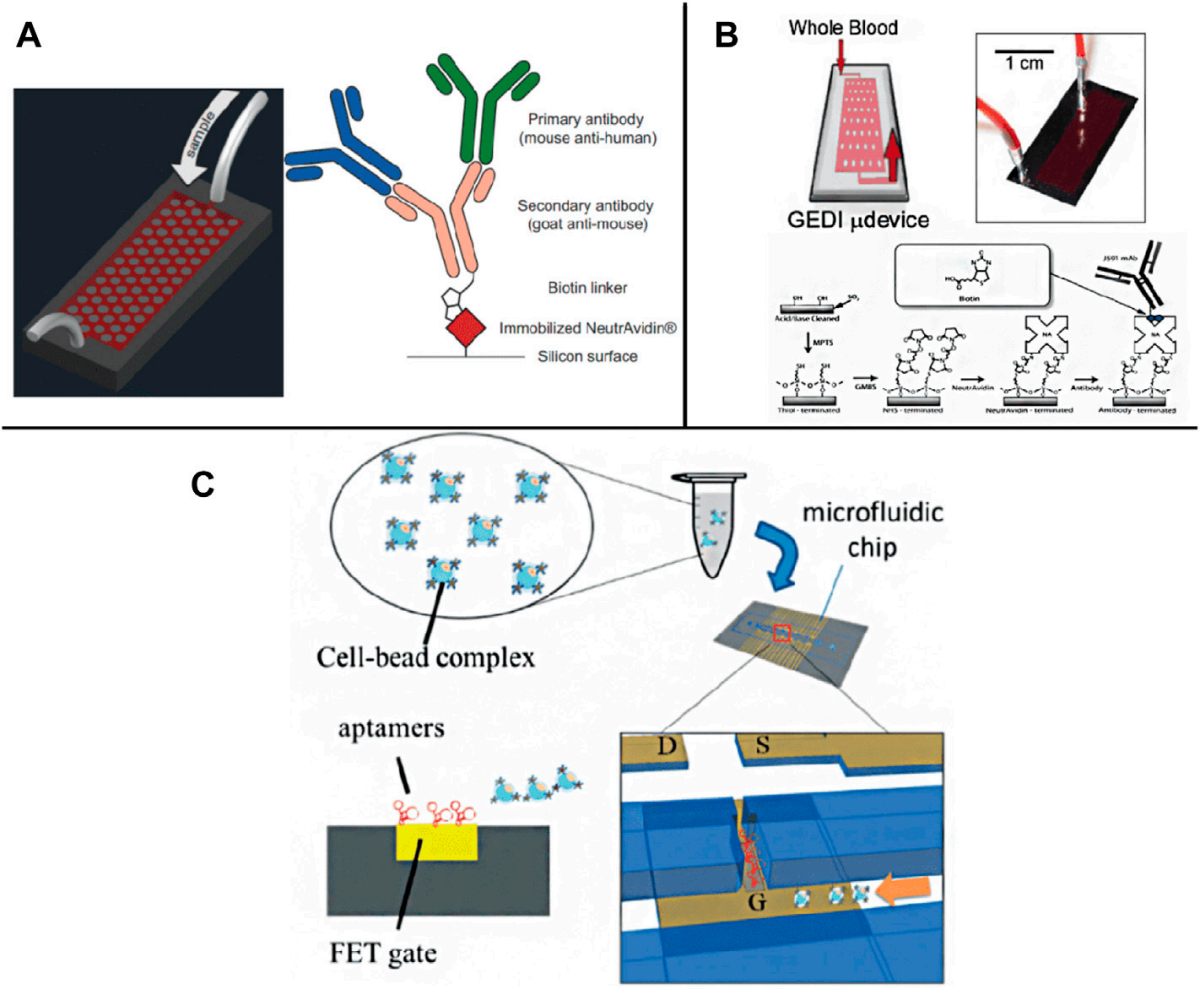
Schematic illustration of membrane antigen and aptamer recognition based chips. **(A)** A GEDI device combined EpCAM and MUC1 detection (Thege et al., 2014); **(B)** A GEDI microfluidic device for whole blood detection (Kirby et al., 2012); **(C)** A FET chip for CTC trapping by bonding CTCs to specific aptamer (Chen et al., 2019).

In addition to the EpCAM specific recognition, other approaches based on tissue-specific membrane antigens, such as prostate-specific membrane antigen (PSMA) of prostate cancer and epidermal growth factor receptor 2 (HER2) of breast cancer, have been designed to uniquely separate tissue-specific tumor cells from blood (Santana et al., 2012). In 2010, the Kirby group developed a geometrically enhanced differential immune capture chip (GEDI), used to separate prostate CTCs using PSMA. Its purity is higher than EpCAM coated CTCs chip and opens up a stage for capturing CTCs using tissue-specific antibodies other than EpCAM (Gleghorn et al., 2010). Subsequently, the group combined EpCAM and mucin 1 (MUC1) in a GEDI (Figure 2A), which was shown to be more effective than a single marker (Thege et al., 2014). Different antibodies or antibody mixtures used to capture CTCs can obtain populations that a single capture ligand may miss, so this method is becoming increasing popular. It is reported that another geometrically enhanced differential immune capture (GEDI) microfluidic platform designed by Kirby et al. can promote the collision frequency between CTCs and antibody functionalized microcolumn and reduce nonspecific leukocyte adhesion to enhance the enrichment of CTCs (Kirby et al., 2012) (Figure 2B). In this platform, prostate-specific membrane antigen (PSMA) introduces cross barriers to effect size-dependent cell trajectories to increase capture opportunities. The results showed that the capture efficiency of the GEDI microfluidic platform coated with anti PSMA was 97 ± 3%.

##### 2.1.1.3 Aptamer Recognition

Compared to antibodies, the aptamer is a small oligonucleotide (such as DNA, RNA and polypeptide), which is easy to bind to molecular and cellular components (Song et al., 2019). They can also be used to specifically recognize target molecules on the cell surface and further integrate into CTCs capture (Bai et al., 2014; Wu L. et al., 2019; Wu et al., 2020b,). Systematically evolved through the exponential enrichment (SELEX) process, the ligands are easy to synthesize and separate. They have high sensitivity and specificity, which is the key feature of aptamers for separating CTCs (Zamay et al., 2019). For example, Lee Group has developed a new microfluidic platform that integrates field-effect transistors (FETs) and chambers for automatic detection and counting of CTCs (Figure 2C). Only target cells bound to a specific aptamer on the FET sensor array can be enumerated (Chen Y. H. et al., 2019). In addition, aptamer functionalized nanostructures were introduced to increase the topographic interaction between targeted CTCs and the surface of specific antibody coating to restore the separation of CTCs. It has satisfactory capture efficiency (Cai et al., 2017; Yu et al., 2019; Fraser et al., 2019).

Although the positive separation method of biological affinity can separate CTCs with a high efficiency and purity, due to the heterogeneous expression of specific surface markers, the ability to target CTCs is limited, and the potentially important CTCs subsets are lost. In addition, affinity-based strategies require sufficient time to prepare samples and sufficient interaction between cells and antibodies, resulting in reduced cell viability and throughput.

#### 2.1.2 Enrichment Based on Physical Screening

According to the differences between CTCs and other blood cells in physical properties such as cell size, density, charge, and deformation ability, CTCs can be screened without biomarkers. Compared with the biological affinity method, the experimental operation of the physical screening method is often simpler without chemical modification and biomarkers; therefore, it has little effect on cell activity. Physical screening methods include active separation methods using external physical fields, such as dielectrophoresis (Augustsson et al., 2012; Alshareef et al., 2013; Aghaamoo et al., 2019), surface acoustic wave (Antfolk et al., 2015; Magnusson et al., 2017), optical tweezers technology (Hu et al., 2019), and passive separation methods with or without external force intervention using inertial effect (Tanaka et al., 2012; Sollier et al., 2014; Warkiani et al., 2014) and viscoelastic effect (Tian et al., 2018; Lim H. et al., 2019) in microscale hydrodynamics.

##### 2.1.2.1 Active Separation Methods

**FIGURE 3:**
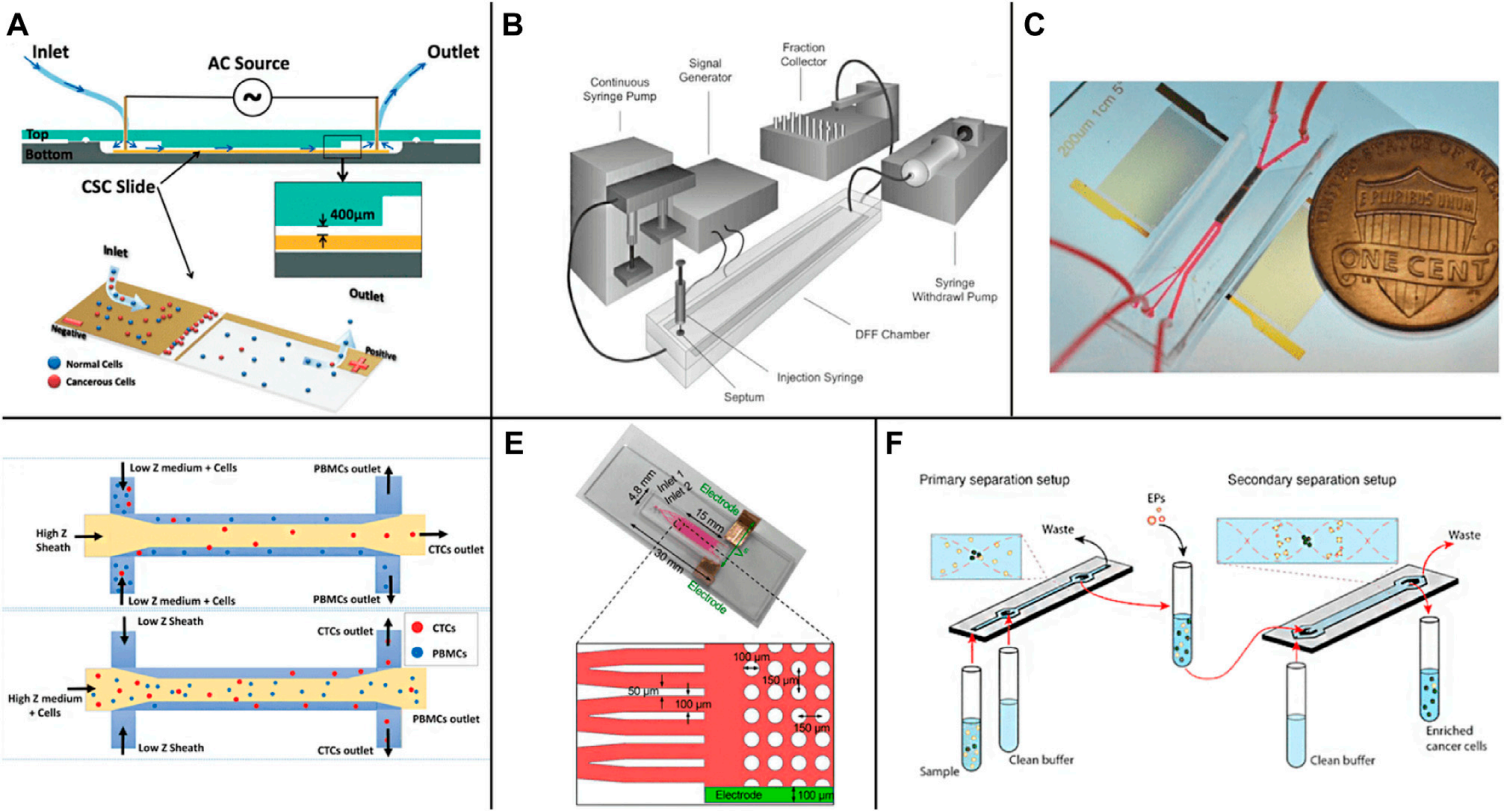
Active enrichment methods based on physical screening **(A)** The CTCs detection chip based on AC electric field (Jahangiri et al., 2020); **(B)** The DEP flow fractionation (depFFF) configuration in CTCs detection (Gascoyne et al., 2009); **(C)** The acoustic CTC separation device (Wu et al., 2018); **(D)** Size insensitive isolation of CTCs from PBMCs (Karthick et al., 2018); **(E)** The microfluidic device on a glass slide with anti-EpCAM modification on the micropost in the device (Sun et al., 2019); **(F)** The two-step acoustophoresis (Undvall Anand et al., 2021).

The active separation mainly separates CTCs by imposing an external field source manipulating cells. The interaction between dielectric particles and the electric field (Dielectrophoresis, DEP) can be used for cell separation, sorting, and capture (Kwizera et al., 2021). Due to the difference in dielectric properties, such as polarization constant between different kinds of cells, cells can be distinguished by applying an appropriate electric field. Jahangiri et al. (2020) completed the separation of CTCs and blood cells from different kinds of breast cancer by applying a low-frequency alternating current (AC) electric field on the chip (Figure 3A). Gascoyne et al. (2009) separated and screened CTCs in blood under the condition of an external AC electric field (Figure 3B), realized the enrichment of CTCs, and obtained more than 90% cell capture rate with a fast processing time and much higher separation efficiency than biological affinity method. Montoya et al. developed a label-free dielectrophoresis microfluidic platform to promote the enrichment of circulating hybrid cells (CHCs) in a high-throughput and rapid manner by consuming healthy peripheral blood mononuclear cells (PBMC). 75% of the clinical samples were enriched, which proved that this method is a promising non-invasive method for analyzing tumor cells of patients (Montoya Mira et al., 2021).

Manipulating cells, droplets, and particles by sound waves in microfluidic chips is a rapidly developing field that is widely used in cell and particle sorting, blood separation, droplet transportation, and rare or cancer cell enrichment (Yiannacou and Sariola, 2021). Wu et al. (2018) realized the sorting of CTCs in peripheral blood by using surface acoustic waves (Figure 3C), adding sound field and surface acoustic wave sensor according to the arrangement of cells with different sizes, densities, and shapes in the standing wave field. The recovery rate of more than 86% CTCs can be obtained at the flux of 7.5 ml/h. Karthick et al. reported a combined sorting method based on acoustic impedance contrast and cell size for separating CTCs from peripheral blood mononuclear cells (PBMC) using acoustic electrophoresis in a microchannel (Figure 3D). By controlling the acoustic impedance contrast of the liquid in the channel, the CTCs whose acoustic impedance is higher or lower than PBMC are isolated. Hela and MDA-MB-231 cells were isolated unlabeled from PBMC (collected from 2.0 ml of blood) within 1 h, and >86% recovery and >50 times enrichment was obtained (Karthick et al., 2018). Sun et al. realized the efficient detection of CTCs in human blood by creating a separate capture area and flow area in the microfluidic device (zone chip) (Figure 3E) and using the patterned dielectrophoresis force to guide the cells from the flow area to the capture area (Sun et al., 2019). Antfolk et al. Introduced a simple cell separation instrument based on acoustic electrophoresis for continuous flow, unbiased and label-free separation of cancer cells and leukocytes based on acoustic electrophoresis. The cells were acoustically pre-aligned using ultrasound before separation, while the cells were maintained in the initial suspension medium. Even if the transverse displacement of particles in the sound field is less than 50 μm, the platform can still separate cells and particles with high precision. The system can separate the particles directly in the suspension medium without matching the acoustic characteristics of the sample with the system of multiple laminar flows (Antfolk et al., 2015). Undvall et al. presented a new two-step acoustic electrophoresis (A2) method for separating unfixed live cancer cells from whole blood lysed by red blood cells (RBC) (Figure 3F). The method uses the initial acoustic flow pre-separation step to separate the cells according to the acoustic mobility of the cells (Undvall Anand et al., 2021). Magnusson et al. used a clinical scale acoustic microfluidic platform to enrich paraformaldehyde-fixed or living cancer cells. The platform can be adjusted to meet the requirements of high cancer cell recovery or higher purity and can process 5 ml of blood in about 2 h. It opens up a broader field for the post-separation analysis and characterization of CTCs in patient samples in the future (Magnusson et al., 2017).

Using tumor cell targeting molecules to bind homologous red blood cells (RBC) to tumor cells shows a significant difference in optical constants (size and average refractive index) between red blood cell-bound CTCs and other blood cells. Then, the modified CTCs can be accurately separated under laser irradiation in the optical jet system. Experiments showed that CTCs effectively modified with red blood cells were finally separated from blood with high purity (more than 92%) and high recovery (more than 90%). Throughout the process, CTCs were shown to maintain membrane and functional integrity. This method provides a convenient tool for early diagnosis and treatment monitoring of cancer, which performs well in the non-invasive and accurate separation of CTCs (Hu et al., 2019).

##### 2.1.2.2 Passive Separation Methods

**FIGURE 4:**
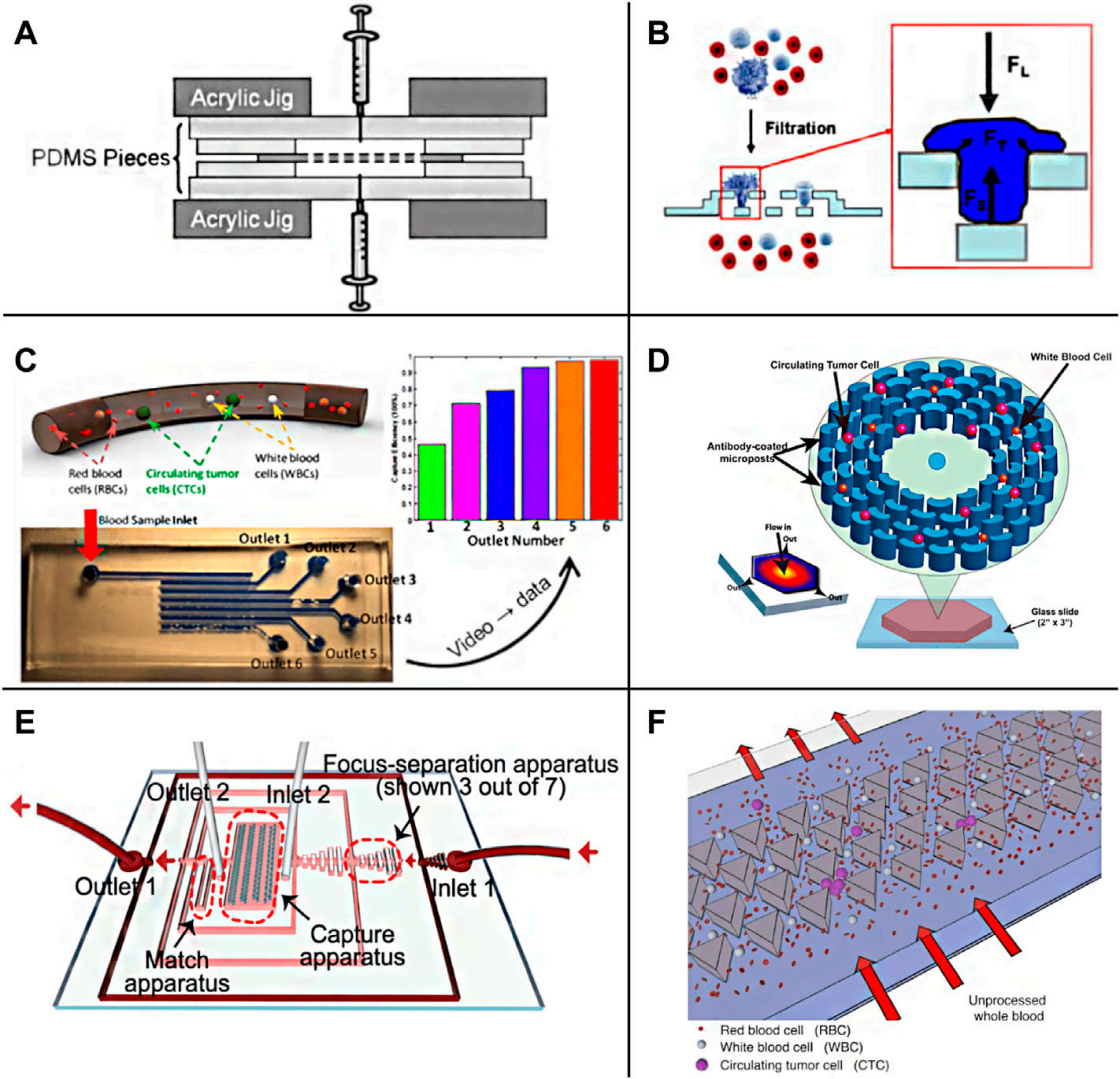
Passive enrichment methods based on physical screening **(A)** A functional microdevice consists of parylene membrane filter (Lin et al., 2010); **(B)** Filtration process and forces on a trapped cell of the three-dimensional microfiltration device (Zheng et al., 2011); **(C)** Sequential Size-Based Microfluidic Chip (Ren et al., 2018); **(D)** The OncoBean Chip (Murlidhar et al., 2014); **(E)** A schematic illustration of the chip combining microscale hydrodynamics and pore sieve principle (Lu et al., 2020); **(F)** Schematic representation of the Cluster-Chip operation (Sarioglu et al., 2015).

Despite the external field source method having high efficiency in cell separation, it is difficult to integrate it into the chip, so Lin et al. (Lin et al., 2010) designed a straightforward CTCs separation chip according to the principle of pore screen filtration (Figure 4A). By adjusting the size of the filter hole, the separation of CTCs can be completed according to the difference in cell size, and the recovery rate is more than 90%. Zheng et al. reported a new three-dimensional microfiltration device (Figure 4B), which can enrich living circulating tumor cells from the blood. The device consists of two layers of palling film, and the holes and gaps are accurately defined by lithography technology. The position of the hole moves between the top and bottom membranes. The bottom membrane supports the captured cells, minimizes the stress concentration on the cell membrane, and maintains cell viability during filtration (Zheng et al., 2011). After that, various functional microfluidic platforms have been optimized to enhance the isolation of CTCs. Ren et al. (2018) designed a high-throughput CTCs capture chip according to cell size and deformability (Figure 4C). The chip has multiple channels, and the channels are connected by multiple rows of miniature shrink tubes, in which there is a capture cavity designed according to the size of CTCs. When the blood flows through the intersection of the main channel and the miniature systolic tube, the capillary action generated by the surface tension will drive the fluid through the systolic tube. At this time, CTCs will be trapped in the capture chamber, and other components in the blood can pass smoothly and then enter the adjacent channel. In order to ensure a high capture rate of CTCs, the process can be repeated between multiple channels, and the capture rate of CTCs could exceed 95%. Chen et al. (2021) constructed a pore sieve chip system based on the biomimetic splenic sinus microstructure. The filter hole of the fissure structure has lower flow resistance than the traditional circular structure. Through the optimization of flow velocity and slit width, it can ensure the high cell activity of CTCs while realizing efficient separation. However, because the size of leukocytes and CTCs is the same, the screening accuracy of this method is low, which may produce false-positive results. In addition, setting a filter hole on the cell flow path is easy to increase the negative pressure due to blockage, which affects the separation efficiency. Qin et al. (2015) used the resettable cell trap (RCT) mechanism to separate cells by using an adjustable pore size that can be removed regularly to prevent blockage according to the size and deformability of cells. Inspired by the antifouling membrane, Kim et al. (2017) used an independent onboard laboratory system equipped with fluid-assisted separation technology (fast) to separate live CTCs from whole blood without prior sample processing. Numerical simulation and experiments show that under 1 kPa, this method provides uniform, non-clogging, and ultrafast cell enrichment, and the pressure drop is much lower than the traditional size-based filtration.

There are usually many physical barrier microstructures in the chip screened for CTCs according to the cell size, and the processing of these precise structures is often difficult. With the development of fluid mechanics theory on a micro-scale, based on the study of particle motion law in microfluid, the separation and enrichment of CTCs can be realized just by regulating the fluid without any microstructure. A microfluidic separation device with the asymmetric bifurcation of laminar flow around obstacles is used to separate particles. Particles determine their path based on their size. All particles of a given size follow an equivalent migration path to obtain high resolution (Huang et al., 2004). Murlidhar et al. reported an ultra-high throughput oncogene chip (Figure 4D) that can isolate live CTCs even at a high screening rate. The platform uses radial flow to produce different shear on the chip so that CTCs can be effectively captured under a high flow rate (Murlidhar et al., 2014). The results showed that at a high flow rate of 10 ml/h, the recovery rate of rare CTCs (cancer cell lines MCF7 and h1650) was 93%, and the capture efficiency was more than 80%, which increased the chance of CTCs recovery in subsequent downstream analysis. Ahmed et al. (2017) described a new device, a size-controlled immune capture chip, for efficient, sensitive, and spatial resolution CTCs capture and detection. The size-controlled immune capture chip can make CTCs interact selectively, frequently, and widely with the surface of immunocoated microcolumn optimized by fluid dynamics. CTCs with different antigen expression levels can be effectively captured and spatially resolved around the microcolumn. The capture efficiency is greater than 92%, and the purity is 82%. Wu et al. (2020c) designed a fluid multivalent nano interface and decorated the microfluidic chip with aptamer functionalized leukocyte membrane nanovesicles to efficiently separate CTCs. This fluid biomimetic nano interface with an active supplement combination provides significant affinity enhancement of four orders of magnitude and shows seven times the capture efficiency compared with the monovalent aptamer functional chip in blood. At the same time, this soft nano interface inherits the biological advantages of natural biofilm, minimizes the adsorption of background blood cells, and maintains good CTCs activity (97.6%). Kulasinghe et al. (2019) designed a square channel chip with a straightforward structure based on the inertial effect on the micro-scale. Due to the action of the dean and the center point of the square pipe, the vortex will gather preferentially near the center point of the large diameter pipe. Therefore, CTCs will be enriched near the center of the channel while other components are arranged on the outside. There is no microstructure or capture cavity in the chip to increase the negative pressure, so there can be a higher breakthrough in the processing flux. In addition, without the influence of any other external effects, the cells can better retain their physiological activity and morphological characteristics. Lim S. B. et al. (2019) also introduced tangential flow at the T-shaped channel to screen the blood cells close to the tube wall according to the inertial effect to realize the separation of CTCs. Zhang et al. (2018) and Tian et al. (2018) used the viscoelastic effect to complete the separation of CTCs. Using the fluid with low viscosity and no shear thinning can achieve an effect similar to the inertial effect. Moreover, the theoretical convergence model of this method is relatively simple, which is convenient for more accurate numerical simulation analysis. Zhu Z. et al. (2021) used a polymer film as material and constructed spiral microfluidic chips of trapezoidal channels through jigsaw puzzle technology. Using the combined action of inertial force and Dean eddy current in trapezoidal channel, the separation of CTCs can be realized at a high flux of 3 ml/min, and the experimental recovery rate is 90%–94%. The microfluidic chip system designed by Lu et al. (2020) combines microscale hydrodynamics and the pore sieve principle (Figure 4E). First, the preliminary separation of blood cells is completed through the inertial effect, and then the capture of CTCs is realized by the triangular microcolumn array. Ensuring a high capture rate of 94.8%, the flux can reach 40 ml/h. Liu Z. et al. (2021) combined filtration with deterministic lateral displacement (DLD) and designed a cascaded DLD microcolumn array chip to achieve 96% recovery of CTCs at a high throughput of 1 ml/min and eliminate 99.99% white blood cells. Cancer cells metastasize in the blood in single migrating circulating tumor cells (CTC) or multicellular clusters (CTC clusters). Sarioglu et al. Designed a chip with a triangular micro column structure to sort CTCs clusters with high precision (Figure 4F). When the CTCs cluster flows through the triangular microcolumn, it will be captured at its apex due to the intercellular connection, and a single cell will pass directly along the side waist surface of the microcolumn. This method requires that the flow rate should not be too high; otherwise, the large shear force will destroy the structure of the CTCs cluster, resulting in capture failure. The chip was used to separate the actual samples, determine the heterogeneity of CTCs clusters, and find that they may contain tumor-associated macrophages (TAMs), which is of great significance for the study of the interaction between TAMs and CTCs (Sarioglu et al., 2015).The label-free physical screening strategy is characterized by the CTCs integrity and fast sample processing. However, due to the heterogeneity of CTCs and the overlapping of CTCs and background cells, the observed recovery and purity of targeted CTCs are not satisfactory.

#### 2.1.3 Enrichment by Combining Biological Affinity With Physical Screening

**FIGURE 5:**
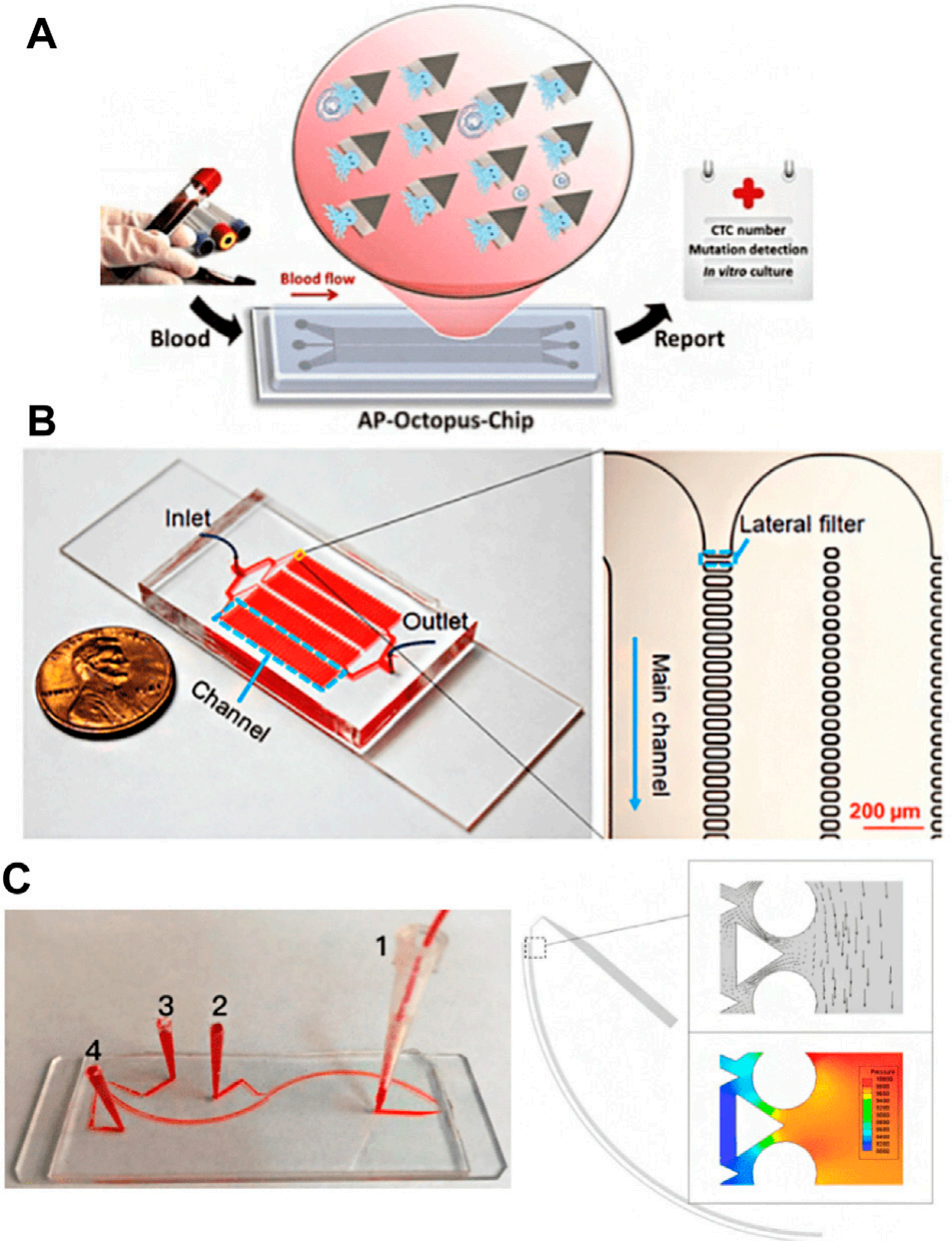
CTCs Enrichment by combining biological affinity with physical screening **(A)** The Octopus chip for cell capture (Song et al., 2019); **(B)** The LFAM device consisting of four serpentine main channels (Chen et al., 2019); **(C)** The antibody functional microsphere integrated filter chip (Su et al., 2019).

CTCs separation chips based on biological affinity and physical screening methods have advantages. The former is more specific, while the latter has higher separation efficiency. However, both of them also have shortcomings. For example, the biological affinity method depends on exogenous markers, which often affect the cell activity of CTCs, while the physical screening method has low separation accuracy and is easy to produce false-positive results. Therefore, the researchers tried to combine the two methods (Pei et al 2019; Lee, et al., 2017), effectively remove leukocytes using a label-free method, and then separate CTCs with high efficiency and purity using an affinity-based method. Song et al. (2019) designed a deterministic lateral displacement (DLD) pattern microfluidic chip modified with multivalent aptamer functionalized nanospheres (AuNP-syl3c) based on the principle of deterministic lateral displacement (Figure 5A). When cells with different sizes and elasticity flow through the angular triangular microcolumn array area, they choose different paths due to the collision with the microcolumn. By adjusting the size and spacing of microcolumn, CTCs can produce lateral displacement when colliding with microcolumn, and other blood cells flow out along the original path. In addition, the microcolumn is modified with AuNP-syl3c, the multivalent aptamer antigen-binding efficiency is increased by 100 times, and the capture performance of CTCs is significantly improved. Compared with the monovalent nucleic acid aptamer modified chip, the capture efficiency of this method is increased by more than 3 times. Chen K. et al. (2019) used the chip with lateral microcolumn array structure, modified EpCAM antibody on the microcolumn (Figure 5B), combined size based separation with immunoaffinity based separation to improve the capture efficiency of CTCs and reduce the nonspecific geometric capture of normal cells. Using the same principle, Su et al. (2019) developed a functional antibody microsphere integrated microchip (Figure 5C) by integrating cell size and tumor cell surface-specific antigen and introducing surface-functionalized modified zinc oxide microsphere, which greatly improved the effective capture area.

### 2.2 Negative Enrichment

Although high capture purity can be obtained by positive selection of CTCs by affinity methods such as anti-EpCAM, anti PSMA, and aptamer due to the specificity of tumor markers, the main disadvantage of this technology is that it may not be conducive to the integrity and vitality of CTCs recovered in the separation process. Therefore, the negative enrichment or selection of CTCs has been widely used. Due to the small size difference between leukocytes and CTCs, leukocytes are often the most interfering factor in sorting CTCs in blood. Therefore, leukocytes can be selectively isolated to achieve the purpose of CTCs enrichment, that is, reverse enrichment (Jiang et al., 2021). Hematopoietic cells were removed by negative selection targeting antigens (CD45) that do not express CTCs (Sun et al., 2018; Tan et al., 2018). The recovered high-purity CTCs were trapped on the platform surface, while most other blood cells were washed away. The reverse enrichment strategy can not only effectively realize the separation of CTCs; moreover, CTCs whose EpCAM expression is down-regulated due to EMT, even non-epithelial tumor cells, can be enriched. At the same time, it can also avoid the effect of direct labeling on the activity of CTCs cells.

**FIGURE 6:**
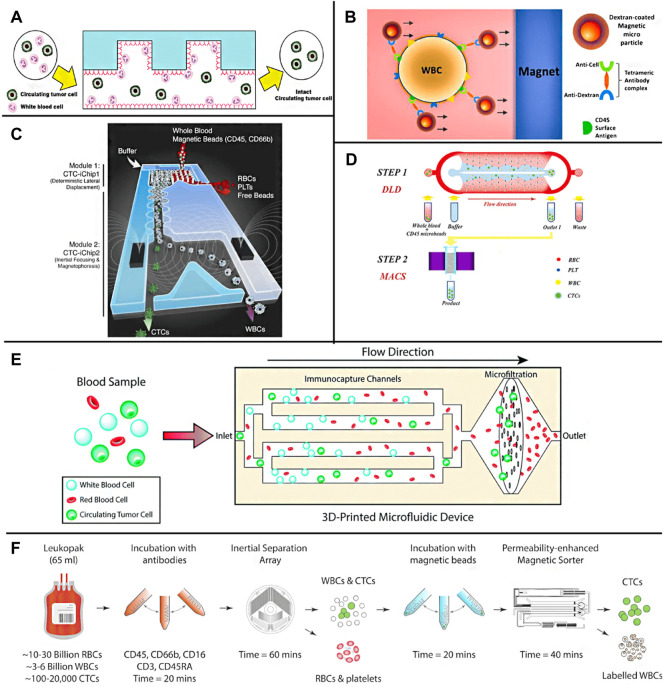
Negative enrichment methods for CTCs **(A)** Pictorial presentation of negative enrichment of the GASI chip (Hyun et al., 2013); **(B)** Illustration of immunomagnetic WBC depletion in which magnetic particles bound to WBC through Tetrameric antibody complex in whole human blood (Sajay et al., 2014); **(C)** The CTC-iChip schematic (Karabacak et al., 2014); **(D)** The capture platform integrating a DLD structure with a MACS separator for inline operation (Wang et al., 2019); **(E)** A schematic showing the tumor cell enrichment process in the 3D-printed microfluidic device (Chu et al., 2019); **(F)** A schematics illustrating the microfluidic approach for untouched CTCs isolation from leukapheresis products (Mishra et al., 2020).

Liu et al. used the commercial easysep system to deplete leukocytes through magnetic nanoparticles and tetramer antibody complexes targeting CD45 and then collected rare CTCs from peripheral blood samples of cancer patients (Liu et al., 2011). According to the results of low cell analysis, capture efficiencies of 56% (47 of 84 samples) and 53% (17 of 32 samples) were obtained from patients with cancer and melanoma, respectively. In contrast, flexible microfluidic platforms (e.g., microfluidic magnetically activated cell sorters (MACS) and CTCs-iChip) have been introduced for negative capture (Giordano et al., 2012; Ramirez et al., 2019; Cheng et al., 2019). In addition, the negative selection of the microfluidic platform can isolate CTCs with no or less EpCAM expression, while the recovered CTCs are complete and have relatively good survivability. It is reported that geometrically activated surface interaction chips can improve the capture efficiency of labeled leukocytes by enhancing the interaction between leukocytes and the chip surface (Hyun et al., 2013) (Figure 6A). In addition, Sajay et al. Proposed an upstream immunomagnetic removal technology to remove CD45^+^ labeled leukocytes and then use a specially designed micro fabrication filter membrane to remove chemical-free erythrocytes and separate targeted CTCs (Sajay et al., 2014) (Figure 6B). The results showed that about 90% of targeting MCF-7 and NCIH 1975 cells could restore blood samples at the peak. Karabacak et al. (2014) designed an integrated dual-chip separation system (Figure 6C). Firstly, leukocytes and CTCs were quickly separated from the blood through two physical screening methods of DLD and inertial effect, and then magnetic beads modified with CD45 and CD66b composite antibodies were used to bind leukocytes selectively, and then the accurate screening of leukocytes and CTCs was realized under the induction of external magnetic field. Ensuring a high capture rate of CTCs, this method can also achieve high flux, and it only takes 2 h for 8 ml blood samples processing. Wang et al. (2019) used a similar DLD-MACS method to analyze the clinical samples of patients with liver cancer (Figure 6D). The capture rate of CTCs under the flow rate of 60 μl/min is 85.1% ± 3.2%, and the experiment shows that this method still has a good separation effect for tumor cells with low expression of EpCAM. Chu et al. (2019) developed an integral 3D microfluidic device (Figure 6E), which combines immune removal and post-filtration to enrich CTCs directly from whole blood negatively. Blood samples first flow through the immune capture area modified with CD45 antibody, selectively screen out leukocytes, and then pass through 3 μM pore size filter membrane to remove the small volume of red blood cells and platelets to realize the separation of CTCs. The CTCs separation chip designed by Mishra et al. (2020) combines immunomagnetic separation with inertial effect (Figure 6F) and uses immunomagnetic beads modified with a variety of antibodies to label leukocytes. Under the external strengthening of the magnetic field and fluid regulation, it can realize the efficient separation of CTCs and leukocytes. The enrichment of CTCs can reach 105 times, and the flux is up to 168 ml/h. Overall, the advantage of the negative enrichment strategy over positive selection is that the rate of recovered CTCs is higher. However, the separation purity is usually lower than that of the positive method, requiring multiple separation processes (Kang et al., 2019; Civelekoglu et al., 2022).

For the negative selection of CTCs (strategy based on biological affinity), it usually has a recovery rate of more than 90%. However, the purity of recovered CTCs is less than 10% because some background cells are not labeled by antibodies against leukocytes surface markers and avoid subsequent depletion.

## 3 On-Chip Detection of CTCs

**FIGURE 7:**
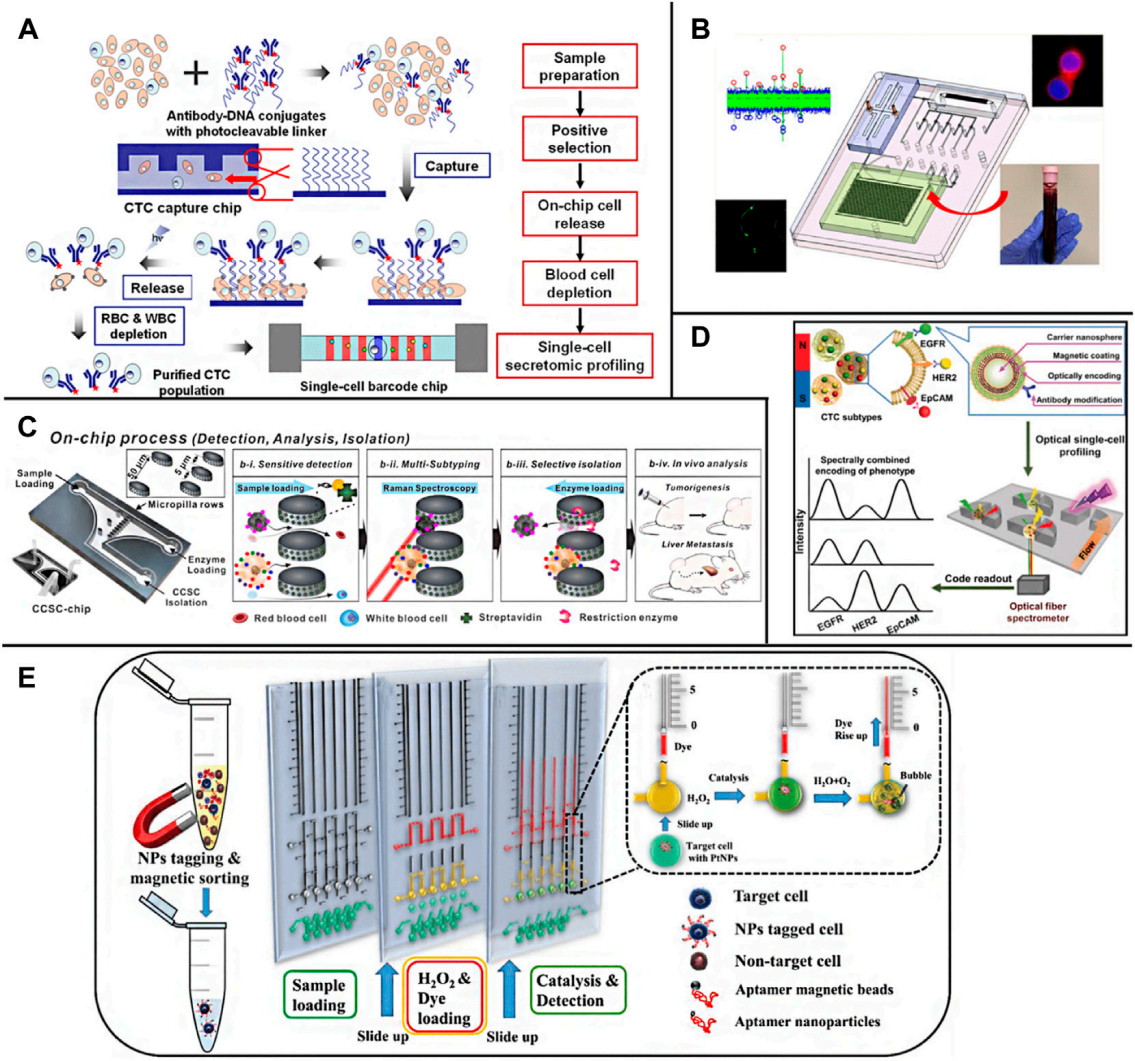
On-chip detection of CTCs **(A)** Overall strategy for CTC isolation and single-cell secretome analysis (Deng et al., 2014); **(B)** A system modularity chip for the analysis of rare targets (SMART-Chip) (Pahattuge et al., 2021); **(C)** A 3-step process for blood sample solutions with RANs labeled CCSCs and CTCs (Cho et al., 2018); **(D)** A multifunctional nanosphere-mediated microfluidic platform for multiplex biomarker profiling of heterogeneous CTCs (Wu et al., 2020); **(E)** The working principle of the aptamer-conjugated PtNPs with volumetric bar-chart chip readout for quantifiable visual detection of CTCs (Abate et al., 2019)^.^

For the detection of CTCs, cell staining is usually carried out first and then observed by a fluorescence microscope (Qian et al., 2015). However, the sensitivity of this method needs to be improved, and the reproducibility is poor, which requires manual operation and manual counting. In recent years, researchers have optimized and improved the imaging analysis method of CTCs on-chip. Watanabe et al. (2014) developed a cell capture platform using on-chip sorting (on-chip Biotechnology) and used anti-CD45 coated magnetic beads to negatively enrich remove leukocytes, and then fixed and labeled the samples. Then, the enriched and labeled samples were sorted according to the expression of cytokeratin, vimentin, and CD45. The captured cells were immediately subjected to genome-wide amplification, followed by a mutation analysis using deep targeted sequencing and copy number analysis using quantitative polymerase chain reaction (qPCR). Deng et al. (2014) developed an integrated microfluidic system specially used to simplify the separation, purification, and single-cell secretory omics analysis of whole blood CTCs (Figure 7A). The first is to capture CTCs through antibody conjugate encoded by photodegradable single-stranded DNA and microfluidic chip producing fretting protein. The captured CTCs are then photochemically released from the chip by brief ultraviolet irradiation and then negatively consume red blood cells (RBC) and white blood cells (WBC). The high-purity CTCs are then delivered to the single-cell barcode chip (SCBC), which integrates the enhanced polylysine (PLL) barcode mode and can capture a very small number of target cells on the chip. A single CTC is isolated in a microchamber and used to analyze a group of functional proteins secreted by a single CTC. The microfluidic system can process 1 ml of whole blood samples in less than 2 h, and the separation efficiency is more than 70%. The platform can also classify CTCs into specific phenotypes through the characteristics of surface markers and conduct single-cell secretory omics analysis on these subsets. Watanabe et al. (2018) successfully stained the CTCs of patients with metastatic non-small cell lung cancer isolated by a chip with a fluorescent-labeled antibody targeting tumor cell markers. The desktop on-chip cell sorter is equipped with disposable microfluidic equipment to detect and isolate rare tumor cells for subsequent molecular analysis. Wang et al. (2020) used immune microspheres modified with CD45 antibody to label leukocytes. After a wedge chip completes the preliminary sorting of blood cells, CTCs can be directly distinguished under bright-field microscopic imaging, and automatic counting can be realized through image processing software. Lee et al. (Lee and Kwak, 2020), through multi-channel fluorescence imaging, simultaneously characterizing the expression of estrogen receptor (ER), progesterone receptor (PR), and human epidermal growth factor receptor 2 (HER2) on CTCs, completes the rapid diagnosis and typing of breast cancer. Wang J. et al. (2021)) introduced a gas driving device into the integrated CTCs separation, immunofluorescence staining, and imaging system, which greatly reduced the time and reagent consumption and was able to capture and recognize CTCs within 90 min. The smart chip developed by Pahattuge et al. (2021) integrates CTCs sorting, cell counting, and immunofluorescence imaging modules, which realizes the fully automatic operation of the separation and detection of CTCs in blood and avoids human interference **(** Figure 7B).

In addition, some common spectral detection methods, represented by fluorescence spectroscopy, are also widely used in the detection of on-chip CTCs. Shen et al. (2018) designed a chip based on the gold film, sorted CTCs by immunomagnetic separation, and then observed and detected CTCs by near-infrared fluorescence method based on surface plasmon resonance. Because the light absorption and spontaneous fluorescence intensity of the biological sample matrix in the near-infrared region are very small, and the fluorescence signal intensity is greatly enhanced by the surface plasmon resonance effect, the detection sensitivity of this method is nearly 10 times higher than that of ordinary fluorescence analysis method. Cho H. et al. (2018); Cho H. Y. et al. (2018) used gold nanoparticles modified with antibodies and Raman signal molecules to label CTCs (Figure 7C). The CTCs captured on the chip can be characterized and detected *in situ* by surface-enhanced Raman technology. This method has high sensitivity and can distinguish common CTCs and circulating tumor stem cells (CTSCs) according to the difference in Raman signal peaks. Dhar et al. (2018) wrapped the CTCs obtained after inertial vortex separation in the droplets containing matrix metalloproteinase (MMP) reaction system through droplet microfluidic technology. Due to the high MMP reaction activity of the target CTCs, the detection and counting of CTCs can be realized by the resonance fluorescence transfer phenomenon generated by the enzymatic reaction system. Wu et al. (2020d) labeled the target protein on CTCs with magnetic nanoparticles loaded with antibody and fluorescence coding at the same time, captured CTCs with a chip under an external magnetic field (Figure 7D), and characterized the contents of epidermal growth factor receptor (EGFR), HER2 and EP-CAM on a single CTC through fluorescence intensity.

In addition to optical analysis methods, researchers also realized the direct digital reading or visual analysis of CTCs chip detection results by using sensing elements. Chen Y. H. et al. (2019) used Al-GaN/GaN with high electron mobility as the material to make field-effect transistors (FETs), set the FETs sensor array on the chip, and modify the specifically recognized nucleic acid aptamer of EpCAM on its surface, to realize the continuous capture and counting of CTCs. The high transconductance gain of FETs makes the bioelectronic sensor have high detection sensitivity. Experiments show that this method can realize the rapid and automatic detection of CTCs in a wide dynamic range and provide accurate cell count data. Niitsu et al. (2018) first proposed a fully integrated CMOS circuit based on a vector network analyzer and transmission line for CTCs and exosome analysis and detection. A fully integrated architecture is introduced to eliminate unwanted parasitic components and achieve high sensitivity to analyze very low concentrations of CTCs in blood. Kim et al. (2021) introduced a one-time intelligent microfluidic platform called “DIS-μ Chip”, in which microfluidic flow sensors are integrated. Due to integration, the flow between the flow sensor and the microfluidic function is significantly reduced without a pipeline connection. Isolated CTCs from the blood of patients with pancreatic cancer using the DIS-μ chip can further be extracted the cancer-specific gene information by the digital droplet PCR, proving that the DIS-μ chip is effective. Chang et al. (2015) isolated and detected CTCs from blood samples using a microchip system integrating immunomagnetic, high-throughput fluidics, and size-based filtration. Magnetic beads with antibody functionalization were used to target CTCs in samples. Then, the mixture passes through a micro-machined chip fluid chamber containing an 8 μM-diameter aperture array. The fluid runs parallel to the microchip and generates a magnetic field below it, pulling the beads and cells bound to it to the surface of the chip to detect CTCs larger than pore diameter and remove free beads and other smaller particles bound to it. The system allows high volumetric flow rate detection and allows the sample fluid to circulate multiple times through the system in a short time. An average of 89% MCF-7 of breast cancer cells was detected. Gao et al. (Gao et al., 2018) combined CTCs chip screened by composite immunomagnetic beads with droplet digital PCR chip to improve the detection sensitivity through PCR amplification. Abate et al. (2019) added Pt nanoparticles (Pt NPs) modified with nucleic acid aptamer to the blood sample to be tested, obtained the target CTCs bound with Pt NPs through immunomagnetic separation, and introduced them into the detection chip pre-loaded with H_2_O_2_ and dye. Because Pt can catalyze the decomposition of H_2_O_2_, the generated oxygen gas will lead to the rise of the dye liquid column, to the content of CTCs in the blood can be judged according to the height of the liquid column (Figure 7E). Jian et al. (2020) labeled CTCs with magnetic metal-organic frameworks (MOF) nanoparticles modified with glucose oxidase. After the TiO_2_ nanotube array on the chip captured CTCs by magnetic force, Fe^2+^/Fe^3+^ in MOF was reduced to FeO through the photocatalytic action of TiO_2_ and the electrochemical signal was obtained by differential pulse voltammetry to realize the quantitative detection of CTCs.

## 4 Clinical Application Prospect and Challenge of CTCs Microfluidic Biosensor

CTCs have attracted extensive attention in tumor research as a new tumor biomarker, and their clinical application is also being widely studied. The emerging microfluidic technology has become a general tool for basic and applied tumor metastasis research because of its relatively low cost, simple operation, small volume, and accurate fluid control. With the progress of micromachining technology, the microfluidic platform is thriving. The functional microfluidic platform will contribute to an in-depth understanding of cancer biology and multiple drug screening. Based on the large amount of information provided by CTCs, the use of CTCs includes early screening and cancer diagnosis, treatment and drug resistance monitoring, drug evaluation, disease progression, and prognosis. It is reported that a detectable tumor lesion contains at least 109 tumor cells (Steeg, 2006; Lawson et al., 2015). Although high-resolution imaging techniques such as CT, PET and MRI (Wang Q. et al., 2021) have been widely used in the clinical detection of tumor lesions, their ability to detect early tumor formation is limited. The detection of non-invasive CTCs has been explored for early tumor screening and diagnosis (Ilie et al., 2014). It can detect early events before the formation of primary tumors (Pantel and Alix-Panabières, 2010; Alix-Panabières and Pantel, 2016; Zhang et al., 2016; Austin et al., 2018; Moon et al., 2018; Poudineh et al., 2018), which provides a basis for early cancer detection (Nagrath et al., 2007; Zahirović et al., 2022).In addition, CTCs count is helpful to predict tumor progression and overall survival (Gorin et al., 2017). The level of CTCs in many cancer patients is highly correlated with tumor progression. CTCs Count is helpful to identify tumor progression. For patients with breast cancer who had a CTCs count below 5 CTCs/mL in blood, the overall survival rate of patients with a CTCs count over 5 CTCs/mL was lower (Krebs et al., 2010; Krebs et al., 2011). In addition, the phenotype of CTCs is related to the tumor stage (Jordan et al., 2016), which can be used as a good indicator to evaluate tumor progression. CTCs can provide information about tumor progression before and during treatment and provide information about the molecular evolution of tumor cells during treatment (Kidess-Sigal et al., 2016). For example, as cancer patients develop resistance to treatment, CTCs show more mesenchymal-like CTCs (Yu et al., 2013). CTCs analysis was used to determine the maximum tolerated dose and guide the optimal dose selection of anticancer drugs (An et al., 2021; Guo et al., 2021). Through the simultaneous monitoring of EMT biomarkers and apoptosis of CTCs, the results show that even if they act on the same tumor type, there are significant differences in the optimal drug dose, indicating that tumor heterogeneity has an impact on the drug use of patients (Pei et al., 2020b). CTCs counts can monitor treatment and help clinicians make the best chemotherapy decisions. CTCs count decreases with the continuation of effective treatment. CTCs count may become an effective tool to monitor the early effect of cancer treatment (Sheng et al., 2014). Tracking and detecting the CTCs of patients in real-time during the treatment can predict the treatment results faster and more accurately, which can be used to evaluate the efficacy of clinical drugs (Bruna et al., 2016; Stevens et al., 2016; Praharaj et al., 2018), and customize the treatment scheme for individual patients (Weinstein et al., 2018; Kozminsky et al., 2019).

At the same time, in the transformation and application of clinical tumor diagnosis and treatment, the detection of tumor biomarkers of CTCs still faces many challenges: first, the content of tumor biomarkers in the blood is rare, and there is tumor heterogeneity, which makes it very difficult to isolate and purify them. To overcome this challenge, it is necessary to develop a microfluidic platform that fully uses the physical and biological characteristics of tumor biomarkers. Secondly, the high-precision and fully automated systems based on microfluidic separation and purification of tumor biomarkers are mostly in the scientific research stage and have not been widely used in the clinic. Moreover, the integration between circulating tumor biomarkers and the microfluidic platform needs to be improved. In order to further promote the development of the microfluidic system with high sensitivity and good repeatability, a large number of clinical trials need to be carried out on a variety of cancer patients. In addition, our understanding of tumor biology is still in its infancy. With the in-depth study of the role of various circulating tumor biomarkers in tumor formation and development, more powerful and effective commercial microfluidic systems will emerge as the times require.

## 5 Conclusion

In this paper, we reviewed the separation strategy, technical principle, and research progress of microfluidic chip separation of CTCs. The separation strategy can be divided into positive enrichment and negative enrichment. The technical principle is also mainly divided into biological affinity and physical screening. At the same time, the main technical methods and optimization strategies of CTCs on-chip detection are introduced. The application prospect of microfluidic chips in tumor diagnosis and treatment and the development direction of microfluidic chips in tumor detection are analyzed. With the rapid development of microfluidic chip technology, its ability for microscale fluid manipulation, microstructure processing, and integrated sensing and detection has been greatly improved, which further promotes the development of CTCs separation microfluidic chip technology. Using a microfluidic chip as a platform to separate and detect CTCs in peripheral blood can give full play to the advantages of micro, high efficiency, easy automation, and integration of the chip itself, and finally realize the rapid and accurate analysis of CTCs in clinical blood. It has important application space in many fields such as early tumor diagnosis, recurrence, metastasis monitoring, and anti-tumor drug evaluation (Ankeny et al., 2016; Pei et al., 2020; Sun et al., 2021; Kelley et al., 2014; Farshchi and Hasanzadeh, 2021; Dong et al., 2019).

Although microfluidic devices have successfully achieved the capture performance of CTCs through various affinity-based or label-free methods, no method has satisfactory separation results of high efficiency through porosity, purity, recovery, and throughput at the same time. At this stage, the CTCs chip still has great room for improvement in screening accuracy and screening efficiency. In response to this challenge, because it is difficult to have both accuracy and efficiency, future chip design should focus more on realizing a single goal. On the one hand, we should focus on improving the cell purity and the cell activity of CTCs screening for basic research. Firstly, separate the blood roughly using the inertia effect and screen out the larger white blood cells and CTCs. Then, screen CTCs accurately adopting droplet sorting (Joensson and Andersson Svahn, 2012; Dong and Fang, 2020) through immunomagnetic separation (Chen et al., 2013). Droplet sorting technology can achieve the accuracy of single-cell analysis, which has been reported for tumor cell screening (Popova et al., 2019). On the other hand, in clinical testing, the researchers focus to realize the high-throughput analysis of clinical samples. The electrical analysis method can be used to set the appropriate threshold according to the difference in specific membrane capacitance and cytoplasmic conductivity of different kinds of cells to realize the rapid analysis of CTCs passing through the detection window (Lannin et al., 2016; Chiu et al., 2017; Zhao et al., 2018; Zhang Y. et al., 2019). In addition, microfluidic chip technology belongs to an interdisciplinary field. The development of CTCs chips also benefits from technological breakthroughs in fields such as microelectromechanical systems (MEMS), materials science, hydrodynamics, and biomedicine. In the future, the microfluidic platform designed for multi-step CTCs separation will be integrated with advanced functions to minimize the shortcomings of the complex sample preparation process. With the development of related technologies, the CTCs chip is expected to become an important platform for basic tumor research and early clinical diagnosis of cancer in the future.
